# Quantifying the need for enhanced case management for TB patients as part of TB cohort audit in the North West of England: a descriptive study

**DOI:** 10.1186/s12889-017-4892-5

**Published:** 2017-11-15

**Authors:** Angela Tucker, Jeniffer Mithoo, Paul Cleary, Mark Woodhead, Peter MacPherson, Tom Wingfield, Stefanie Davies, Carolyn Wake, Paddy McMaster, S. Bertel Squire

**Affiliations:** 1 Department of Clinical Sciences Liverpool School of Tropical Medicine, Liverpool, UK; 2 Centre for Epidemiology, University of Manchester, Manchester, UK; 3 University of Liverpool, Liverpool, UK; 4 Public Health England Field Epidemiology Service North West, Liverpool, UK; 5 Department of Respiratory Medicine, Central Manchester University Hospitals NHS Foundation Trust, Manchester, UK; 6 Faculty of Medical and Human Sciences, University of Manchester, Manchester, UK; 7 Department of Social Medicine, Karolinska Institutet, Stockholm, Sweden; 8 North West Public Health England Centre, Liverpool, UK; 9 Institute of Infection and Global Health, University of Liverpool, Liverpool, UK; 10 Tropical and Infectious Diseases Unit, Royal Liverpool and Broadgreen University Hospitals, Liverpool, UK; 11 Paediatric Infectious Diseases, Pennine Acute Hospitals Trust, Bury, UK

**Keywords:** Tuberculosis, TB, Enhanced case management, ECM, Cohort audit, Cohort review, Social factors, Treatment completion

## Abstract

**Background:**

Patients with TB have diverse and often challenging clinical and social needs that may hamper successful treatment outcomes. Understanding the need for additional support during treatment (enhanced case management, or ECM) is important for workforce capacity planning. North West England TB Cohort Audit (TBCA) has introduced a 4-level ECM classification system (ECM 0–3) to quantify the need for ECM in the region. This study describes the data from the first 2 years of ECM classification.

**Methods:**

Data collected between April 2013 and July 2015 were used to analyse the proportions of patients allocated to each ECM level and the prevalence of social and clinical factors indicating need for ECM. Single variable and multivariable logistic regression models were constructed to examine the association between ECM level and treatment outcome.

**Results:**

Of 1714 notified cases 99.8% were assigned an ECM level: 31% ECM1, 19% ECM2 and 14% ECM3. The most common factors indicating need for ECM were language barriers (20.3%) and clinical complexity (16.9%). 1342/1493 (89.9%) of drug-sensitive, non-CNS cases completed treatment within 12 months. Patients in ECM2 and 3 were less likely to complete treatment at 12 months than patients in ECM0 (adjusted OR 0.47 [95% CI 0.27–0.84] and 0.23 [0.13–0.41] respectively).

**Conclusions:**

Use of TBCA to quantify different levels of need for ECM is feasible and has demonstrated that social and clinical complexity is common in the region. Results will inform regional workforce planning and assist development of innovative methods to improve treatment outcomes in these vulnerable groups.

**Electronic supplementary material:**

The online version of this article (10.1186/s12889-017-4892-5) contains supplementary material, which is available to authorized users.

## Background

Tuberculosis (TB) is an infectious airborne disease of major global public health concern. It is estimated that in 2015 there were 10.4 million incident cases of TB resulting in 1.4 million deaths worldwide [1]. In England TB incidence has declined by one third over the past 4 years to a rate of 10.5 per 100,000 [2]. Despite this reduction the TB notification rate in England remains higher than in most other Western European countries [3] and more than three times higher than in the USA [4]. Public Health England has declared TB to be a national public health priority area [5]. In the North West of England, although overall TB notification rates are relatively low (7.9 per 100,000 in 2015), rates are high in urban centres such as Manchester (27 per 100,000 3 year average 2013–2015) [2].

TB Cohort Audit (TBCA), sometimes referred to as Cohort Review, is a systematic multidisciplinary review of the management and outcomes of all TB cases in a geographical area with the aim of improving clinical and public health outcomes and thereby strengthening the prevention and control of TB [6, 7]. The National Institute for Health and Care Excellence (NICE) has recommended that TBCA should be included in TB prevention and control programmes since 2012 [8]. TBCA was first introduced in the UK in London in 2010 and was implemented in the North West in 2012 due to pockets of high incidence in regional urban centres. North West TBCA meetings bring together TB nurses, TB physicians, public health professionals, epidemiologists and analysts to review and discuss the management of each individual TB case. Cases are reviewed approximately 6–9 months following treatment commencement, at or near the end of a standard course of treatment. The process captures detailed quantitative data and monitors outcome indicators. In addition, presentation and discussion of cases in a supportive environment provides a forum for training and education of staff and for the identification of issues relating to case management, workforce or quality of care.

Completion of TB treatment is imperative from both a clinical and public health perspective with failure to complete increasing the risk of relapse, death, drug resistance and disease transmission [9]. Although treatment is highly effective and relatively inexpensive, the minimum treatment duration of 6 months for non-resistant cases can make it difficult for patients to fully adhere to the regimen [10]. Standard Case Management (SCM) coordinated by a named case manager, most often a specialist TB nurse, is appropriate for non-clinically complex patients able to self-medicate and have monthly follow-up in a hospital or community setting [11]. Cases with clinically and/or socially complex needs require a higher level of support to ensure successful treatment outcomes and for this reason the National Institute for Health and Care Excellence (NICE) and the Royal College of Nursing (RCN) recommend that such cases should receive Enhanced Case Management (ECM) [11, 12]. ECM is defined by NICE as care that “is provided when someone has clinically or socially complex needs…As part of ECM, the need for Directly Observed Treatment (DOT) is considered, in conjunction with a package of supportive care tailored to the person’s needs.” (8) These additional care needs are commonly delivered by the named case manager, usually the TB nurse, working alongside a specialist multidisciplinary TB team.

Prior to the introduction of TBCA in the North West of England there was no comprehensive way of quantifying the need for ECM in the area. NICE recommend that TBCA should be used to collect data on the numbers requiring ECM in order to inform local needs assessments [12]. Quantification of these needs within a TB population will be important for workforce planning, particularly relating to TB nurse staffing levels. For this reason, in 2013, following the pilot of TBCA in the North West, a series of enhanced case management levels was introduced into TBCA ranging from level 0 (standard case management) to level 3 (highest level of enhanced case management) (Table 1). Patients now undergo a standardised risk assessment and are assigned an ECM level based on their clinical and social needs and hence the level of input they required from the multidisciplinary team. This study aimed to quantify the ECM levels of patients in the region, evaluate the prevalence of the underlying social and clinical factors indicating need for ECM, and examine the association between ECM level and treatment outcome.

**Table 1:** Key definitions of variables used in TB Cohort Audit

Enhanced Case Management	“Enhanced Case Management is provided when someone has clinically or socially complex needs. It commences as soon as TB is suspected. As part of ECM, the need for Directly Observed Treatment (DOT) is considered, in conjunction with a package of supportive care tailored to the person’s needs.” (12)
ECM level 0	Equivalent to Standard Case Management: no clinical or social issues impacting on treatment, ability to self-medicate and have monthly follow-up in a hospital or community setting, no complex contact tracing requirements
ECM level 1	Clinical and/or social issues impacting on treatment and necessitating fortnightly visits
ECM level 2	Complex clinical and/or social issues impacting on treatment and necessitating weekly visits
ECM level 3	Very complex clinical and/or social issues impacting on treatment. May require directly observed treatment (DOT)
Clinically complex	Clinically complex cases are those with one or more of: renal impairment, HIV co-infection, diabetes, drug resistance or severe side-effects
Hard to reach group (HTRG)	Children, young people and adults whose social circumstances or lifestyle, or those of their parents or carers, make it difficult to: recognise the clinical onset of TB, access diagnostic and treatment services, self-administer treatment or attend regular appointments for clinical follow-up

## Methods

### Data source

Data collected from North West TBCA between April 2013, representing the introduction of the ECM classification system into TBCA, and July 2015 were combined with routine data from Public Health England’s Enhanced TB Surveillance (ETS) system. All notified TB cases were reviewed at TBCA in this period and were eligible for inclusion in the analysis. Cases that had not been assigned an ECM level were excluded from the analysis.

### Development of enhanced case management (ECM) levels

Definitions of ECM levels 0–3 were agreed based on expert consensus between representatives from clinical medicine, nursing and public health with ECM 0 representing SCM and ECM levels 1–3 representing different levels of ECM (Table 1). A guide was produced to assist TB nurses in allocating patients to appropriate ECM levels (Appendix) which aimed to reflect the diverse nature of patient needs and the impact these have on patient management. The ECM levels were piloted as part of TBCA, discussed and refined at TBCA meetings and subsequently finalised by the North West TBCA Steering Committee. The ECM classification system was formally introduced into TBCA in April 2013. Risk factors for Enhanced Case Management are evaluated by TB nurses at the beginning of treatment to establish the level of support the patient will require from the multi-disciplinary team and the ECM level is formalised and agreed at the time of TBCA.

As part of ECM classification, data were collected on specific factors indicating need for ECM including language barrier, clinical complexity (which includes those cases with renal impairment, HIV coinfection, diabetes, drug resistance or severe side effects), being in a hard-to-reach group (HTRG), non-adherence to treatment, homelessness, alcohol dependency, drug dependency, imprisonment, previous TB diagnosis and mental health problems. Some key definitions used in TBCA for ECM levels and factors indicating need for ECM are given in Table 1.

### Statistical analysis

We performed a descriptive analysis to assess the proportion of TB cases classified to each ECM level and examine the prevalence of the recorded clinical and social indicators for ECM. We then examined the association between ECM level and treatment completion at 12 months. Single and multivariable logistic regression analyses were performed and multivariable models were adjusted a priori for age group and gender followed by other demographic factors found to be significantly associated with ECM level. Cases with one or more drug resistance, CNS disease and post-mortem diagnoses were excluded from the analysis as these cases would not be expected to complete treatment within 12 months. The analysis was repeated after excluding all patients with a language barrier documented as this is a major indicator for ECM in the North West but this may not be the case in other areas where demographic characteristics of patients may differ and/or the TB nurses have language skills relevant to the population served. All statistical analyses were performed using Stata, Version 12 (Stata Corp., College Station, Texas).

### Ethics

This study used anonymised routinely collected public health audit and surveillance data and individual participant assent was not sought. The North West TB Summit Steering Committee formally reviewed and approved the study protocol.

## Results

### The population

During the study period, there were 1714 TB cases reviewed at TBCA of whom 1711 (99.8%) had been assigned an ECM level and were therefore included in the analysis. The median age of included cases was 40 years (interquartile range 28–57 years) and 999/1711 (58.4%) of cases were male (Table 2). The majority of cases were non-UK born (1077/1675, 64.3%) but amongst those in ECM level 3 the majority of cases were UK born (128/241, 54.7%). Those of Pakistani and white ethnicity accounted for over 60% of cases (564/1652 (34.1%) and 457/1652 (27.7%) respectively). Patients living in the most deprived national index of multiple deprivation (IMD) quintile accounted for 1027/1625 (63.2%) of cases and those living in the least deprived quintile accounted for 92/1625 (5.7%) of cases. Nearly half the cases had pulmonary disease (814/1711, 47.6%), of whom 335 (41%) were sputum smear positive.

**Table 2:** Demographic characteristics of TB cases in the North West of England by Enhanced Case Management level *N* = 1711

Independent variable	Total (%)	ECM Level				*P*-value*
		0 *N* = 616 (36%)	1 *N* = 528 (31%)	2 *N* = 326 (19%)	3 *N* = 241 (14%)	
Age group (*n* = 1710^a^)						<0.001
0–15	89 (5.2)	10 (1.6)	34 (6.5)	28 (8.6)	17 (7.1)	
16–30	434 (25.4)	167 (27.1)	124 (23.5)	92 (28.2)	51 (21.2)	
31–45	485 (28.4)	193 (31.3)	150 (28.5)	79 (24.2)	63 (26.1)	
46–65	394 (23.0)	136 (22.1)	122 (23.2)	67 (20.6)	69 (28.6)	
> 65	308 (18.0)	110 (17.9)	97 (18.4)	60 (18.4)	41 (17.0)	
Gender (*n* = 1711)						0.85
Male	999 (58.4)	359 (58.3)	313 (59.3)	187 (57.4)	140 (58.1)	
Female	712 (41.6)	257 (41.7)	215 (40.7)	139 (42.6)	101 (41.9)	
Born in the UK (*n* = 1675^a^)						<0.001
No	1077 (64.3)	427 (71.2)	349 (67.4)	195 (60.4)	106 (45.3)	
Yes	598 (35.7)	173 (28.8)	169 (32.6)	128 (39.6)	128 (54.7)	
Ethnic group (*n* = 1652^a^)						<0.001
White	457 (27.7)	143 (24.2)	118 (23.1)	91 (28.8)	105 (44.9)	
Pakistani	564 (34.1)	212 (35.8)	204 (40.0)	93 (29.4)	55 (23.5)	
Indian	251 (15.2)	112 (18.9)	49 (9.6)	62 (19.6)	28 (12.0)	
Black African	198 (12.0)	65 (11.0)	71 (13.9)	37 (11.7)	25 (10.7)	
Bangladeshi	42 (2.5)	17 (2.9)	19 (3.7)	4 (1.3)	2 (0.9)	
Other^b^	140 (8.5)	43 (7.3)	49 (9.6)	29 (9.2)	19 (8.1)	
Deprivation quintile (*n* = 1625^a^)						<0.001
1 (least deprived)	92 (5.7)	46 (7.8)	21 (4.2)	12 (3.9)	13 (5.6)	
2	120 (7.4)	64 (10.8)	26 (5.3)	19 (6.2)	11 (4.7)	
3	148 (9.1)	67 (11.3)	44 (8.9)	16 (5.2)	21 (9.1)	
4	238 (14.7)	96 (16.2)	65 (13.1)	44 (14.3)	33 (14.2)	
5 (most deprived)	1027 (63.2)	318 (53.8)	339 (68.5)	216 (70.4)	154 (66.4)	

^a^Missing data

^b^Includes Black Caribbean, Black Other, Chinese and Mixed/Other

^*^P-value for differences across categories of ECM: Chi-square test for trend (dichotomous variables), Pearson’s Chi-square test (other categorical variables)

### ECM classification and factors indicating need for ECM

1095/1711 (64%) patients were assessed as requiring some level of Enhanced Case Management with 528/1711 (31%) classified as level 1, 326/1711 (19%) as level 2 and 241/1711 (14%) as level 3. Level 0, or Standard Case Management, accounted for 616/1711 (36%) of all cases (Table 2). Language barrier was the most common recorded factor indicating need for ECM (348/1711, 20.3%) followed by clinical complexity (289/1711, 16.9%), non-adherence to treatment (119/1711, 7.0%), previous TB diagnosis (95/1711, 5.6%), being from a HTRG (66/1711, 3.9%), and mental health problems (58/1711, 3.4%) (Table 3). Homelessness (45/1711, 2.6%), injecting drug use (50/1711, 2.9%), problem alcohol use (53/1711, 3.1%) and imprisonment (53/1711, 3.1%) were less common overall. The prevalence of all indictors increased with higher ECM level with the exception of language barrier which is most prominent in ECM 1 (Table 3).

**Table 3:** Factors indicating need for ECM by ECM level

Indicator for ECM	Total (%)	ECM level			
		0	1	2	3
Language barrier	348 (20.3)	10 (1.62)	189 (35.8)	96 (29.5)	53 (22.0)
Clinically complex	289 (16.9)	7 (1.1)	70 (13.3)	106 (32.5)	106 (44.0)
Nonadherence to medication	119 (7.0)	2 (0.3)	14 (2.7)	25 (7.7)	78 (32.4)
HTRG	66 (3.9)	1 (0.2)	7 (1.3)	17 (5.2)	41 (17.0)
Previous TB diagnosis	95 (5.6)	27 (4.4)	24 (4.6)	17 (5.2)	27 (11.2)
Mental health problems	58 (3.4)	1 (0.2)	9 (1.7)	16 (4.9)	32 (13.3)
Problem alcohol use	53 (3.1)	2 (0.3)	5 (1.0)	10 (3.1)	36 (14.9)
Imprisonment	53 (3.1)	4 (0.7)	10 (1.9)	12 (3.7)	27 (11.2)
Injecting drug use	50 (2.9)	1 (0.2)	5 (1.0)	13 (4.0)	31 (12.9)
Homelessness	45 (2.6)	2 (0.3)	4 (0.8)	16 (4.9)	23 (9.5)
Drug resistance (one or more)	78 (4.6)	15 (2.4)	18 (3.4)	21 (6.4)	24 (10.0)

### ECM level and treatment outcome

1342/1493 (89.9%) of drug-sensitive, non-CNS cases completed treatment within 12 months: 520/553 (94.0%) in ECM 0, 439/481 (91.3%) in ECM 1, 237/269 (88.1%) in ECM 2 and 146/190 (76.8%) in ECM 3. Increasing ECM level was inversely associated with treatment completion at 12 months (Chi-square test for trend *p* < 0.001). Of drug resistant cases 46/71 (64.8%) completed treatment within 12 months; 10/15 (66.7%) in ECM 0, 16/18 (88.9%) in ECM 1, 15/20 (75.0%) in ECM 2 and 5/18 (27.8%) in ECM 3.

On single variable analysis patients in ECM levels 2 and 3 were significantly less likely to complete treatment within 12 months than patients in ECM level 0 (OR 0.47, 95% CI 0.28–0.78 and OR 0.21, 95% CI 0.13–0.34 respectively). After adjustment for age group, gender, UK born status, deprivation quintile and ethnic group on multivariable analysis patients in ECM 2 and 3 remained significantly less likely to complete treatment in 12 months than patients in ECM 0 (OR 0.47, 95% CI 0.27–0.84 and OR 0.23, 95% CI 0.13–0.41 respectively) (Table 4). When all patients documented as having a language barrier were excluded from the analysis, the association between ECM level and treatment non-completion was strengthened with patients in ECM levels 1, 2 and 3 significantly less likely to complete treatment in 12 months than patients in ECM 0 (Additional file 1 Table S1).

**Table 4:** Association between ECM level and treatment completion within 12 months^a^ (*N* = 1493)

			Single variable analysis		Multivariable analysis	
ECM level	Total	Completed treatment within 12 months ^a^(%)	OR	95% CI	aOR^b^ (95% ci)	95% CI
0	553	520 (94.0)	1		1	
1	481	439 (91.3)	0.66	0.41–1.06	0.67	0.40–1.12
2	269	237 (88.1)	0.47	0.28–0.78	0.47	0.27–0.84
3	190	146 (76.8)	0.21	0.13–0.34	0.23	0.13–0.41

^a^If not drug resistant organism, post-mortem diagnosis or CNS disease

^b^Odds ratios are adjusted for age group, gender, UK born status, deprivation quintile and ethnic group

Chi-square test for trend p < 0.001

## Discussion

The main findings of this study were that: 1. Quantifying the need for ECM using a nurse-led ECM level classification system was feasible as part of TBCA; 2. Social and clinical complexity and the need for ECM of TB patients in the North West was high; 3. More complex patients in higher ECM levels are more likely to have poor treatment outcomes.

TBCA is a process that goes beyond simply evaluating the care of patients; it ensures the follow-up of every TB patient in an area, improves the quality of patient information and enhances patient treatment [7]. In addition, TBCA provides a forum for open discussion of the management of individual TB cases, for obtaining peer support on the management of complex cases, such as those with multiple drug resistance (MDR) or non-adherence, for identification of systemic issues and for further education of staff in TB management and control. TBCA has been very well received by staff in the North West of England where staff have described TBCA as a valuable “community of practice” that has led to mutual respect and a shared sense of purpose between staff from different disciplines [13].

TBCA meetings often involve discussion of the difficulties patients face in completing a long and intensive course of treatment and the challenges staff face in managing complex patients. This, combined with the robust data collection process central to TBCA, meant it was an ideal vehicle to use to begin to understand the complexity of TB cases and the need for ECM in the region. To our knowledge this is the first time that the need for ECM has been comprehensively quantified based on different levels of clinical and social complexity of TB cases. In 2012 Gebril et al. performed a retrospective review of case notes in an inner city cohort in the North West in order to estimate the numbers of TB patients qualifying for ECM. They identified 96/170 (56%) as ECM cases based on medical and/or nursing needs, slightly below our overall ECM estimate of 64% [14]. The more detailed and nuanced ECM classification system combined with the fact that the need for ECM is assessed by the patients’ own TB nurse is likely to account for this difference and is therefore likely to be a more accurate measure of the need for ECM in the area.

The results of this study suggest that social and clinical complexity of TB patients in the North West of England is common and the need for ECM is high, with only 36% assessed as ECM 0 or Standard Case Management (SCM), leaving 64% assessed as requiring some level of ECM of whom 14% required ECM 3 due to very complex clinical and/or social issues impacting on treatment. The Royal College of Nursing and the National Institute for Health and Care Excellence (NICE) have previously recommended at least one whole-time equivalent TB case manager per 20 incident cases requiring enhanced case management and one per 40 incident cases requiring standard case management [8, 11]. Therefore knowledge of the numbers requiring ECM will be important in informing commissioning decisions and ensuring TB prevention and control services are effective in meeting the needs of patients. In addition, as TB incidence in low burden settings is decreasing we are likely to see increasing complexity of patients and we will need to be adept at addressing their needs in order to successfully manage them through to treatment completion and prevention of further transmission. The ECM classification scale will provide a valuable means of monitoring the levels of complexity as the balance begins to shift.

The association between clinical and social complexity, as measured by ECM level, and poor treatment outcome emphasises the importance of having systems in place to effectively manage complex patients. In order for ECM services to be effective, TB nurses need to be equipped with the appropriate skills to effectively manage complex patients and there should be established links and prompt access to allied professionals who can support TB professionals in addressing housing, financial, asylum, immigration, alcohol and drug dependency and other health and social care needs [8, 11]. Directly Observed Treatment (DOT) should also be considered as part of ECM [12] however DOT alone is not necessarily a solution for poor adherence [15] and the appropriateness of providing DOT is a decision that needs to be considered by the clinical team. Such systems are already in place in the North West of England however it is unclear from these data what effect they are already having on treatment outcomes. Further research is warranted to explore in depth the specific challenges patients in these high risk groups face in completing treatment and to determine where improvements can be made in the services currently in place for the management of complex patients in order to improve treatment outcomes in the future.

### Strengths and limitations

This study used data from the national Enhanced TB Surveillance system and from TBCA; robust processes that collect data on all TB cases in the region. Through the TBCA process we were able to obtain data on several clinical and social factors that have not been systematically collected previously. During TBCA a concerted effort is made to optimise the completeness and quality of collected data by amending any inaccuracies as cases are presented.

There were a number of limitations of the study. The ECM level categorisation is a subjective and unvalidated process for measuring the extra support a patient requires from the TB nurse and allocation to ECM level is based on their professional judgement. However, in order to maintain consistency, ECM classifications are discussed at TBCA meetings where reasons for the chosen ECM level are justified. Case complexity is not a clearly defined issue and its definition will inevitably remain in part a subjective process. It will depend on the skills of, and resources available to, the TB case manager; and at a population level it will depend on the characteristics of the population served. For example in the North West, language barrier is a major factor affecting case complexity but this may not be the case in other areas either in terms of the demographics of the population or in terms of the language skills of the TB case managers.

Therefore, although the specific results from the North West of England are not directly generalizable to other regions, the methods used in the North West are potentially generalizable to other areas in the UK and in Europe that have introduced or are considering introducing TBCA as part of their TB prevention and control strategies. The ECM classification system presented can be adapted by service providers to represent the needs of their population and the skills and resources available to the local TB workforce and analysed accordingly. Future work could explore approaches to evaluate case-complexity more objectively and allow comparison between different areas. This may include measuring individual social and clinical factors in more detail and determining their relative importance in influencing both the amount of input required from the TB specialist nurses and treatment outcome using methods such as multivariable analysis or propensity analysis.

ECM scores are representative of the level of clinical and social complexity of patients and we have demonstrated that higher levels of complexity are associated with poorer treatment outcomes. This relationship is likely to be mitigated by the fact that patients in higher ECM levels, by the very nature of the classification, will receive a higher level of input and support from the multidisciplinary team leading to an underestimate of the association between clinical and social complexity and treatment outcome. Conversely, although ECM levels are considered early on in a patient’s treatment, they may be dynamic, evolving with a patient’s needs, and reflect the level of care ultimately required rather than that initially envisaged. Due to the retrospective operational nature of the study and the lack of independent validation of the ECM level assigned there is a possibility that the ECM level reported at the time of TBCA may be influenced, consciously or subconsciously, by knowledge of treatment outcome; potentially leading to an overestimation of the association between clinical and social complexity and treatment outcome.

Finally, the ECM classification system will be useful in informing commissioning decisions, particularly in relation to workforce capacity, however it only reflects cases of active TB and the needs of cases of latent TB infection (LTBI) will also need to be considered in optimising prevention and control services.

## Conclusions

Quantifying the need for ECM through nurse-led classification as part of regional Cohort Audit is feasible and has demonstrated that clinical and social complexity in TB patients in the North West of England and the need for ECM is high. The uses of TBCA go beyond simply auditing performance outcomes and the process can be harnessed to further understand ECM requirements and inform workforce capacity planning, training and service development in the region to improve the management of complex patients in the future.. The ECM classification system could be expanded and adapted to other regions where TBCA has been established as part of TB prevention and control.

## Appendix

**Table 5:** Guide to classification of patients to Enhanced Case Management levels used in TBCA

ECM level	Clinical factors	TB specific	Social factors
0	Physically able to self-medicate No central nervous system impairment Positive IsoScreen at reviews Correct tablet count at reviews	Contact tracing requirements limited to adults in the same household No stigma related issues	No language barriers No housing or finance issues impacting on treatment
1	Elderly to monitor for side effects Children to ensure compliance of child and parent/carer Requires GP or community pharmacy input for blister packs to check correct doses Taking complex medications e.g. HIV medications Disease site e.g. smear positive pulmonary or central nervous system disease	Contact tracing requirements in various areas and/or settings e.g. patient out of area, workplace, community group settings Stigma that can be dealt with through one-to-one education	Requires interpreter for first visit but has some understanding of English Requires signposting for benefits and/or financial issues Patient difficult to reach e.g. no front door bell, more than 1 address, problems getting time off work/college, refusal of home visits
2	Having complex side effects requiring LFT monitoring Needs more regular prompting with medications e.g. blister packs, regular IsoScreen, tablet counts HIV and TB co-infection and starting both anti-retroviral and TB medications at the same time Single drug resistance	Transmission within contacts or children who are contacts Stigma that requires more formal education e.g. through community centres or workplaces	Financial difficulties that may affect treatment compliance e.g. attending clinic, poor nutrition, poor heating Language barriers throughout treatment requiring easily accessible interpreter at each visit either face to face or by phone Alcohol and/or drug dependency without LFT derangement Patient difficult to reach e.g. DNA at clinics, not home for reviews
3	More than one drug resistance Needs reintroduction of medications e.g. due to deranged LFT’s	Complex contact tracing e.g. transmission to children, vulnerable groups, extensive transmission Involvement of PHE for workplace or community screening	Difficult language barriers throughout treatment Homelessness or housing issues due to finance Illegal immigrants, difficulty accessing benefits Potentially dangerous patients where more than one person is required to visit Children who DNA and where social service involvement is required Patient difficult to reach e.g. consistent DNA at clinics, consistently not home for reviews

## Additional file

Additional file 1: Table S1. Association between ECM level and treatment completion within 12 months† excluding patients recorded as having a language barrier (*N* = 1194) (DOCX 12 kb)

## Abbreviations

DNA: Did not attend
ECM: Enhanced case management
HTRG: Hard to reach group
LFT: Liver function tests
NICE: National institute for health and care excellence
PHE: Public Health England
RCN: Royal College of Nursing
TBCA: TB cohort audit
