# Supplementary material for: Quantifying the need for enhanced case management for TB patients as part of TB cohort audit in the North West of England: a descriptive study
Source: BMC Public Health. 2017 Nov 15;17:881. doi: 10.1186/s12889-017-4892-5 (PMC5688729; doi:10.1186/s12889-017-4892-5)
Supplement: Additional file 1: Table S1. — Association between ECM level and treatment completion within 12 months† excluding patients recorded as having a language barrier (N = 1194) (DOCX 12 kb) [file 12889_2017_4892_MOESM1_ESM.docx]

Additional file 1 Table S1 Association between ECM level and treatment completion within 12 months† excluding patients recorded as having a language barrier (N=1194)

|  |  |  | Single variable analysis | | Multivariable analysis | |
| --- | --- | --- | --- | --- | --- | --- |
| ECM level | Total | Completed treatment within 12 months † (%) | OR | 95% CI | aOR‡ (95% ci) | 95% CI |
| 0 | 543 | 511 (94.1) | 1 |  | 1 |  |
| 1 | 311 | 275 (88.4) | 0.48 | 0.29-0.79 | 0.47 | 0.27-0.82 |
| 2 | 190 | 164 (86.3) | 0.40 | 0.23-0.68 | 0.37 | 0.20-0.70 |
| 3 | 150 | 113 (75.3) | 0.19 | 0.11-0.32 | 0.17 | 0.09-0.31 |

†Excluding patients with one or more drug resistance, post-mortem diagnoses , CNS disease and patients with a language barrier
‡Odds ratios are adjusted for age group, gender, UK born status, deprivation quintile and ethnic group
